# The Nursing Supplement

**Published:** 1890-02-08

**Authors:** 


Being the Extra Nursing Supplement of "The Hospital" Newspaper.

All Contributions for this Supplement should be addressed to the Editor, The Hospital, 140, Strand, London, W.C., and should have the word "Nursing" plainly written in left-hand top corner of the envelope.

### En Passant.

THE MIDWIVES' INSTITUTE.—This institute and trained nurses' club has moved to 12, Buckingham Street, Strand, and is open daily from two to half-past eight, or on Fridays, which are lecture nights, till ten p.m. There are now about 200 members, and the club is a growing pleasure to, and a growing power in, the nursing world. The subscription is 5s. annually, and there is a good library of medical books.

CORNWALL NURSES' HOME.—The tenth annnal meeting was held at Truro this month, and Colonel Tremayne presided. The seven nurses have attended sixty-six cases during the last year, some of them gratuitously. The receipts were £695, of which £229 was earned by the nurses. Lady St. Leven was elected president, and Miss Rogers, the lady superintendent, was complimented on her indefatigable work for the institution.

THE BRASSEY HOLIDAY HOME.—This holiday home of trained nurses and others needing rest and sea-air will be opened this spring at Ellenslea Road, St. Leonard's-on-Sea. It is under the patronage of Lord Brassey, and under the management of Sister Frost, who trained at the Manchester Royal Infirmary. The terms will be 15*s.* to a guinea a week. We cannot but hope that steps will be taken to admit certain nurses at 10s. 6d. a-week, or else where are the special advantages of the Home? Many a comfortable sea-side boarding house is open to those who can pay a guinea a week, and there more varied company and more amusement and change can be found.

IN AID OF AN INSTITUTE.—A very successful entertainment, in aid of the Middlesbrough Nurses' Institution, was given in the Town Hall lately to a large and fashionable audience. At the conclusion of the first part of the entertainment the Rev. E. O. Herbert; vicar of St. John's, explained the object of the institution, viz.: "To provide skilled nurses to nurse the sick poor in their own homes." For some time past, Miss Purvis, a lady, and trained, experienced nurse, has been engaged in the work, which proving successful, it is now proposed to make what has hitherto been a limited undertaking to an unlimited one. The vicar, in a spirited speech, appealed to all present for support. We wish every success to those engaged in this work.

THOUSANDS OF POUNDS FOR THE NATIONAL PENSION FUND.—We have the pleasure to acknowledge the following sums towards the £14,000 now being raised to at once bring the Donation Bonus Fund up to £40,000. We hope to be able to acknowledge contributions amounting to £1,000 every week until the £40,000 are completed. Contributions of any amount may be sent to the Editor of The Hospital, or to the Manager, National Pension Fund for Nurses, 8, King Street, Cheapside, London, E.C.:

The Third Thousand.

| | |
|---|---|
| Messrs. Schröder and Co. | £500 |
| Messrs. L. Cohen and Sons | 100 |
| Messrs. Ellis and Co. | 100 |
| Messrs. Ferguson and Clark | 100 |
| Messrs. Foster and Braithwaite | 105 |
| Messrs. Panmure, Gordon and Co. | 100 |

Amount already acknowledged, **Three Thousand Pounds.**

THE "QUEEN."—Last Saturday's *Queen* was a specially interesting number from a nurse's point of view. There was a page of nursing costumes, illustrating the London, Middlesex, St. Saviour's, and other principal hospitals; there was a portrait and life of the late Miss M. B. Hughes, and a portrait and life of Sister Rose Gertrude. The *Queen* constantly gives excellent articles and nursing news now.

SHORT ITEMS.—A lady who is interested in the Haggerston district of London desires to establish a small home for nurses there. She estimates that £200 would be required as capital. Full particulars as to the proposed home may be obtained from Mrs. Franks, late matron of the Kensington Infirmary.—On February 9 sermons will be preached at 11 and 3.30 at the Chapel Royal, Savoy, in aid of the East London Nursing Society.—Lady Hardman has compiled a nursing chart, but we cannot see in what way it is better than those charts already published.—We warn all hospital authorities against Miss Johnstone, who lately decamped from Teignmouth Infirmary leaving the committee considerably in debt. Miss Johnstone applies for nursing posts saying she was trained at St. George's, and has been a sister at Guy's.—Sister Rose Gertrude is the authoress of two religious books for children.

RURAL NURSING ASSOCIATION.—The annual meeting of the Kemerton Branch was held on January 18th, when Sir Richard Temple gave an address on the work of the Association. The rector, the Rev. Jerome Mercier, presided. He said he welcomed the organisation providing trained nursing as a valuable addition to the parish work, and he hoped other parishes would take it up in the same spirit. The report of the year's work showed much good work done, and that the finances of the branch were in a satisfactory state. A thoroughly trained nurse and midwife was at work, and the results were excellent. Sir Richard Temple believed that no one suffered more than the village poor. He was certain that in that favoured district, watered by the Avon and the Severn, there was, nevertheless, a great deal of pain and suffering in every village. It was more specially the women and children who suffered. He asked his audience to conceive what it must be with a long protracted pain of serious illness in the absence of those appliances which they had in the higher classes, and of that assistance which they were able to command. It was impossible for him to find language which adequately pourtrayed the sufferings of the poor in the villages in even ordinary times, to say nothing of the oft-recurring periods of epidemic. What, then, were the remedies? In the first place there was that great remedial process, the Association for Rendering First Aid to the Injured. Further, there was the plan of having trained nurses in country districts. Putting aside those cases of illness and accident of a serious and dangerous nature which could be better treated in a hospital, there was a far larger and even more important class of cases which were not so urgent and not so desperately serious, but, nevertheless, required skilled handling. It is for the benefit of this class the Rural Nursing Association is founded. The nurses go about from cottage to cottage and minister. He was sure such nurses could be found in sufficient numbers if they could only succeed in raising the requisite funds. He believed that such women, with a certain amount of training, would be able to do an incalculable amount of good, and he should certainly say, as an old administrator, that they required something more than mere mental training or manual dexterity. They required women who had sympathetic hearts.

### Introductory Lecture

To the Course Given at the Royal Hants Infirmary, Southampton.

By Percy G. Lewis, M.D., late House Surgeon.

It is one of the most important duties of a modern nurse to be able to give an intelligent report of a case, but it is also that duty which a nurse is longest in acquiring, and which requires, perhaps, more hard work to attain perfection in than anything else which a nurse has to do. Formerly, with the old style of nurse, a doctor thought himself lucky if he could rely on the nurse's statement that the patient had been a "little better" or a "little worse"; now, however, that nurses are taken from a class with more brains, a detailed and accurate report of all that happens in his absence is expected.

I advise all of you who wish to cultivate the power of observation to keep a book wherein, when you go off duty, you may jot down anything you have observed during the day. Begin with only one case, and note everything you can about it. At first, of course, you will put down a good deal of unnecessary matter, and possibly omit that which is most important. Still, don't be discouraged; keep on day after day, and soon you will be able to put down all that is necessary in the fewest words and in the right order.

You have come here to learn nursing, and I presume you all want to get on as quickly as possible. Well, I will give you one golden rule by which you are sure to do this, viz., "When on duty, always be doing something." Nurses are too apt to think that all their work is in the order book, but there can hardly be a greater mistake. When not carrying out orders, "observe," and as an aid to this, talk to, and amuse patients. Many symptoms are not obvious until the patient commences to talk or move, or a delusion which had been carefully hidden may be brought out by a nurse in a little friendly conversation. Patients will often tell a great deal more to a nurse than to a doctor, and a little sympathy will sometimes form very good medicine. You cannot begin your habit of observation too soon. It is best in all cases to observe on a definite plan. It does not matter much whose plan you adopt so long as your observations are done systematically.

I will now give you the method which we use in this Infirmary, just mentioning what kind of things are to be looked out for under each heading.

Your observations should commence directly the patient comes into the ward, or much valuable information may be lost. Divide your observations under three heads:—

*First*, then, is there anything obvious? *e.g.*, Can he walk? If so, does he limp? Can he move both arms? Does he droop one shoulder? Is he bleeding? Does he suffer from shock? Does he smell of alcohol? or of anything? Is he sensible? Can he speak? and if so, is his voice peculiar? Next take his face and special senses. Can he see? Are his pupils large or small? equal or unequal? Is he deaf? Is there any special colour about his face? *e.g.*, cyanosed, pale, or jaundiced. Do you see any difference between the two sides?

*Secondly*, observation must be continued while you are undressing him and putting him to bed. Note the skin as to colour, moisture, dryness, if hot or cold, if any eruptions. It is most important also to note if there are any bruises, and should there be any take care that the medical officer sees them at his next round. Patients, especially in poor-law infirmaries, often bring accusations against nurses, pointing out bruises which they say were inflicted on them in hospital; unless, therefore, they were noted at the time of admission, the accusation would be very difficult to disprove. Note if there are any ruptures or swellings, ulcers or scars, or in fact anything abnormal.

The *third* division of your observations will be done under the headings of the various "systems," as they are called, of the body, commencing always with the organs affected, and as long as the case remains in the hospital you will carry out your observations from day to day in the same manner. *Nervous System:* Under this heading you would note whether there was any delirium or coma, or irritability of manner, trembling, convulsions, wandering, amount of sleep. *Respiratory System:* State of the breathing of regular or irregular, if difficult or spasmodic. If any cough or hœmoptysis. Amount of expectoration. Rate of breathing. *Circulatory System:* If any pain or palpitation in the heart. If any large veins or pulsation in veins. Learn to count the pulse. *Digestive System:* Note the appetite, if pain in the stomach, flatulence, nausea, or vomiting. If any vomit, save it until a doctor can see it, but note if its character changes on being kept, *e.g.*, whether frothy at first and not so after. State of the bowels, character of the stools, which should always be kept in cases of doubt. *Cutaneous System:* Temperature, abnormal dryness or moisture, œdema, rashes, etc., etc. If a wound, amount of discharge, colour, swelling around, etc.

I have not, of course, mentioned everything that you may meet with under the several headings, although I have given you a sufficient number of examples to show the sort of observations you are required to make. The whole of the headings will not always be required; such as are must be mentioned in the order named. A perfect report contains the greatest amount of information in the fewest words, with the absence of all unnecessary information. Although all this seems very simple and obvious, in practice it is only acquired by continually working at it and noting case after case day after day. With regard to your training here, something will be done for you in the way of lectures and teaching in the wards. Still, you will have to depend to a great extent upon yourselves. Get into the way of doing your very best for each patient, put yourself (mentally) in the patient's place, and do what you have to do for him as you would like it done for yourself. A slovenly way of doing things is easily acquired, but very hard indeed to get rid of.

Get into the habit, also, of being on friendly terms with your patients, however disagreeable they may be, without, of course, being familiar with them. Thus you will find them more easy to manage; and the habit will be very useful should you wish to take up private nursing, where obtaining the confidence of your patient is half the battle.

To sum up then, success in nursing means work, work day after day; always do your best, "always, when on duty, be doing something."

### Winchester Training School for Nurses.

The Royal Hants County Hospital is an excellent training school for nurses, and one where they receive every consideration and kindness. It is a hospital where the homelike feeling prevails to a very large extent, and where every nurse, servant, and patient seem equally happy and contented. The porter at the door tells you he has been there twelve years; a ward-maid, who has just left to be married, had been there nine years, and the oldest retainer of the hospital, Mrs. Wise, has been connected with the institution for over twenty years.

"My time is up in three months, and I shall be so sorry!" says a probationer, sighing. "I have been so happy here, and we have ever so many dressings to do, which nurses never have where there is a medical school, and sometimes we have two accidents in a day."

The listener smiles gently at the two accidents a-day, having a vivid remembrance of a bustling London hospital, where accidents arrive hourly, and a constant stream of patients is for ever entering the receiving-room. But then the nurses at Winchester are not run off their legs; they have their two hours of duty daily; they have their five meals a-day, and each has a cubicle to herself. They rise at six a.m., and have prayers at nine p.m., so that the day is no longer than it should be; and they have three weeks holiday in the year. Probationers, after a month's trial, are received for three years' training; they get their certificate at the end of the year, and are then given strings to their caps, and are liable to be sent out to private cases. They receive no payment the first year, but the second and third year they receive £20, with board, lodging, washing, and uniform. The sisters receive £30, and are provided with pretty prune-coloured gowns. A few ladies are received on payment of 30 guineas a-year, or 25 guineas for six months, or 13 guineas for three months. They are treated in every way the same as the nurses.

Lectures are given to the probationers by one of the medical staff, and Mrs. Suckling, the matron, also gives occasional lectures and bandaging classes.

The in-patients to the hospital number about 600 a-year, and the out-patients 1,000. In the last report we see that 2 amputations, 3 osteotomies, 3 hermiotomies, and 2 abdominal sections were performed last year amongst other operations. Ten burns were received, 5 cases of acute rheumatism, 23 fractures, and 10 cases of pneumonia. This proves that in a year a probationer with her wits about her should be able to learn much at the Hants County Hospital.

Then there is a private nursing institution in connection with the hospital which sends out nurses to nearly 200 cases in the year. Also Mrs. Suckling, being well-known, and having established a reputation for her nurses, is often applied to by other institutions in need of trained help. There is, therefore, a future for the nurse who does well at Winchester.

The hospital is in a very healthy situation on the very top of a hill; the sanitary arrangements are excellent, and the wards lofty and never crowded. The committee consider the nurses in every way, as is shown by the fact that they have lately affiliated themselves to the Pension Fund. To sum the matter up in one phrase, the Royal Hants County Hospital is a model training-school for nurses, so long as they do not desire the excitement, rush, and overwork of one of the great London hospitals.

### Examination Questions.

The following is part of the prize answer for last month:—

Burns.—Burns are very likely to die from exhaustion through shock, so the greatest care is necessary in nursing them. A child brought into the ward severely burnt on chest and abdomen, having been previously dressed in the surgery with carron oil or any other dressing the surgeon may have fancied, is immediately put in bed wrapped in a blanket, with hot bottles to the feet and thighs; if very collapsed it has brandy and water every ten minutes, as ordered by the surgeon; if the child is sick it is fed by nutritive enema. It is fed as soon as possible by mouth, at first small quantities at a time, the amount is gradually increased as the child gets stronger, when the most nourishing diet possible is given, not only to build up its physical strength, but to add vigour to its shaken nervous system and to nourish the wounds themselves. Every possible care is taken to prevent the child catching cold; it is very liable to get pneumonia, or kidney mischief, or erysipelas—any septic poisoning in the wounds. If the arm or leg is deeply burnt, hæmorrhage must be looked for, also contraction of the muscles must if possible be prevented, and any stiffening of joints. But the nurse has little to do with the two latter maladies, they come under the surgeon's care.

Cataract.—Patients waiting for this operation should be carefully nursed beforehand; they should be kept as free from worry as possible, well fed, and get plenty of rest. They may take no nourishment for three hours before the operation. They have castor oil the day before, as ordered by the surgeon. After the operation patients are carried back to bed, and kept very quiet in a darkened room. For the first twenty-four hours they usually take only fluids, but this depends very much upon each individual case. The pads must be kept damp and scrupulously clean.

### Everybody's Opinion.

[*Correspondence on all subjects is invited, but we cannot in any way be responsible for the opinions expressed by our correspondents. No communications can be entertained if the name and address of the correspondent is not given, or unless one side of the paper only be written on.*]

One of the Staff writes: Will you allow me to correct a statement which, under the heading of "Central Sick Asylum," appears in this week's Hospital. The sentence objected to runs thus: "The patients enjoyed the concert very much, and so did Nurse Taylor and Nurse Chapman, who have had the nursing of the asylum since its commencement." As a matter of fact the concert was held as usual in Nurse Taylor's ward, and the last-named (Nurse Chapman) has only been attached to the asylum for the last six months, and did not attend the entertainment at all. Nurse Taylor is one of the four senior nurses belonging to the institution, and has for some years past managed most satisfactorily the details consequent upon having a concert-room arranged out of a sick ward. Miss Idabeg and Messrs. A. Wellesley and H. Thorndike were amongst the performers, Miss Maude Chichester, with other artistes, having been prevented from appearing. The insertion of this will greatly oblige.

Two Nurses write: Perhaps it would interest nurses to hear of our midwifery cases among the gipsies. About 12 o'clock one night we were hastily summoned to the small village of tents situated near Boleyn Castle, Plaistow. The one we entered was small and low, with a smouldering fire in the middle, around which several old women sat smoking. The patient laid on a bed of straw covered with a coloured blanket, and appeared to be comforting herself with tea which was boiled on the fire in an ancient tin tea-pot. All the time we were there we sat crouched up on the ground, as the tent would not allow us to stand upright, while the fumes of the coke fire were overpowering. When the old grannie understood we wanted air, she threw back a flap of the tent, so that it was possible to stand up, put our heads out of the top and enjoy the prospect. After waiting a long time a pretty little dark baby came into the world, and was bathed in a bucket which, having stood on the embers to heat water, had got so hot that we had much ado to prevent the baby from being burnt by the edges. At another case we attended, there was an old woman nearly a hundred years old, who caused much amusement by her quaint talk. It was always difficult to move in the tents; the fire being in the middle left us little space. The things required were pushed in under the tent. This was rather awkward, as it was somewhat dark, and an unexpected saucepan of boiling gruel is a little difficult to handle. Large articles were handed down through the open flap. Some of the tents were artistically decorated with pictures slipped between the willow props which supported the structure. We always found the gipsies very grateful for our help, and on one occasion they brought the little brown baby to our church to be baptised.

### Death in our Ranks.

At the London Hospital on January 26th, of double pneumonia, Nurse Agusta Bishop, of the private nursing staff, aged 29.

### Exhibition of Nursing Uniforms.

Some difficulties having arisen with our Scotch friends, the doll nurses have been sent to Shrewsbury, where they will shortly be exhibited. Any institution not yet represented and desiring to join the exhibition, can send the dolls to Miss Oldham, Salop Infirmary, Shrewsbury.

### Appointments.

[It is requested that successful candidates will send a copy of their applications and testimonials, with date of election, to THE EDITOR, The Lodge, Porchester Square, W.]

WEST OF ENGLAND EYE INFIRMARY.—Miss Howard Jones has been appointed matron of this Infirmary at Exeter. Miss Jones trained at St. Bartholomew's, took holiday duty at the "Grosvenor," and then became matron at the Central Ophthalmic for eighteen months. Miss Jones holds excellent testimonials.

SOUTH AFRICA.—Nurse Rose, of Cardiff Infirmary, is shortly sailing for the Cape, to undertake work there as a district midwife.

BRITISH NURSES' ASSOCIATION.—Miss Foggo Thomson has been appointed secretary to this association in place of Miss C. J. Wood, resigned. Miss Foggo Thomson spent a short time at Pendlebury, but is chiefly known as the deliverer of nursing lectures and the wearer of a very pretty uniform. We never heard that Miss Thomson held any certificate.

### Registration of Nurses.

THE British Nurses' Association have just issued a circular letter, with two forms, containing the particulars of their proposed scheme of Voluntary Registration. Nurses will no doubt specially note that in Form No. 1 they are asked to bind themselves and to conform in all respects to the orders of a self-constituted Board, which is practically the Executive Committee of the British Nurses' Association. This is so, because only six of the smaller hospitals are in co-operation with the Executive Committee. We don't believe that nurses will voluntarily consent to take this unnecessary yoke upon them, although it is offered gratis up to March 1st next. From that date half a-guinea will be charged to all nurses who, being tired of their own liberty, may elect to submit themselves to the iron rule of a self-constituted Board of irresponsible censors.

### Notes and Queries.

TO CORRESPONDENTS.—1. Questions may be written on post-cards. 2. Advertisements in disguise are inadmissible. 3. In answering a query please quote the number. 4. If a private answer is desired, a stamped addressed envelope must be forwarded.

NOTICE.—We must really ask our correspondents to be more particular in enclosing name and address. Constantly we receive queries or letters with no name attached.

### Queries.

(49) *Nursing in Australia.*—I should be pleased to have information about work in Australian hospitals.—*V.*

(50) Will any district nurse tell me if, when out-door uniform is provided, *boots* are ever included? I find these in a rough country district a serious item in my expenditure, and certainly think that at least two pair of boots per annum *and an umbrella* should form part of the "out-door uniform."—*A Country District Nurse.*

### Answers.

*L. G.*—(1) The Inman Line of steamers. (2) About two guineas a-week.

*A. L. E.*—"A Handbook of Testing," by Miss Heanly, published by Lewis, price 1s. It is not usual, and it is not right for doctors to address nurses by their surname. Insist on your title of nurse.

*Nurse Alexander.*—It is published by Young J. Pentland, but the price is not mentioned. We should think 2s. 6d.

*Success.*—We cannot advise you upon such a subject, but the scheme sounds promising. It entirely depends upon the amount of support the doctors give you, and vague promises are not to be relied upon. Let us know if you do attempt it.

*E. H.*—The older nurses must trust to the Pension Fund to secure them a cheerful old age. Ask any queries you like, and we will do our best to answer them,

*L. M.* and *J. C.*—You will find all particulars you require in an article, "How to Become a Nurse," in THE HOSPITAL for August 31st, 1889.

(42) *Sister May* will find a complete list of those who have received the R.R.C. in the Monthly Army List.

(48) *M. B.* should apply to the Librarian of the Obstetrical Society of London, Berners Street, W. (51, I think), for the rules relating to the quarterly examination of midwives, which will give her all information. The diploma can be obtained without hospital training, but in this case the midwife must produce evidence that she has attended 25 cases of *midwifery* (not of monthly nursing) to the satisfaction of a qualified medical man, and must also have a certificate stating that she has attended a course of lectures on midwifery, given by a competent doctor.

### Notices.

TO OUR READERS.—In view of certain changes which are imminent in consequence of the large and ever-increasing circulation of THE HOSPITAL, the Editor requests every matron, sister, or nurse who takes the paper regularly to send him notice of her name and address, to The Lodge, Porchester Square, London, W. Postcards may be used.

### Vacancies.

THE following vacancies are announced:

**Matrons.**

Birmingham Orthopædic Hospital; £45.

Croydon General Hospital; £60.

Ilford Village Home Infirmary; £35.

St. Olave's Union, Southwark; £100.

Stockport Infirmary; £60.

**Night-Superintendents.**

Cardiff Infirmary; £28.
Hartshill Infirmary; £30.
Worcester Infirmary; £27.

**Nurses.**

Bangor Nursing Institute; £25.
Beckenham Nursing Home.
Beckett Hospital, Barnsley £20.
Bedfordshire Nurses' Institute; £26
Belgrave Hospital for Children.
Birmingham Orthogradic Hospital.
Blackheath Nursing Institute; £25.
Bolton Infirmary.
Bournemouth Nursing Institute; £25.
Bournemouth Victoria Home; £25.
Bridgewater Infirmary; £25.
Bromley Sick Asylum; £16.
Bristol District Nurses' Society Depot.
Bury St. Edmund's Hospital; £25.
Chelsea Infirmary; £18.
Chester Deaconess House; £24.
Coventry Fever Hospital; £25.
Frome Nurses' Home, £22.
General Nursing Institute.
Hackney Union; £20.
Jersey Nursing Institute; £25.
King's Cross Hospital, Dundee; £22.
Leicester Nursing Institute; £25.
Leith Hospital; £25.
Levick Institution, Paris.
Medical Home, Belsize Park.
Middlesbro' Fever Hospital; £28.
Montreal, Canada; £37.
Mr. Wilson's Institution.
Newcastle Nurses' Home; £22.
Newport Institute.
Notts Nursing Association; £25.
Pendlebury Hospital; £20.
Portsmouth Hospital; £25.
Sheffield Fever Hospital; £25.
Shoreditch Infirmary; £20.
South Hants Infirmary; £30.
St. Cross, Rugby; £25.
St. Leonard's Nurses' Home.
St. Peter's Nursing Institute; £25.
St. Saviour's Infirmary, Dulwich; £20.
St. Vanda's Home, Bromley.
Sunderland Infirmary; £22.
Swansea Nursing Institute; £25—£30.
Tunbridge Wells General Hospital; £25.
Ventnor Consumption Hospital; £20.
Weston-super-Mare Institute.
Whitechapel Infirmary; £20.
Wimbledon Convalescent Hospital; £22.
Wirral Children's Hospital; £23.
Workhouse Infirmary Nursing Association.
Wrexham Infirmary; £20.

**Probationers.**

Great Yarmouth Hospital.
Hospital for Women and Children, Lupus Street, Belgravia.
Medical Home, Belsize Park.
Notts Nursing Association.
Saffron Walden Hospital.
Workhouse Infirmary Nursing Association.

ERRATUM.—Under appointments last week for Miss E. L. Scoan, read Miss E. J. Sloan.

### Amusements and Relaxation.

**FOURTH QUARTERLY WORD COMPETITION**

**Commenced Jan. 4th, 1890, ends March 29th, 1890.**

Three prizes of 15s., 10s., 5s., will be given for the largest number of words derived from the words set for dissection.

Proper names, abbreviations, foreign words, words of less than four letters, and repetitions are barred; plurals, and past and present participles of verbs, are allowed. Nuttall's *Standard* dictionary only to be used.

N.B.—Word dissections must be sent in WEEKLY, arranged alphabetically, with correct total affixed.

The word for dissection for this, the SIXTH week of the quarter, being

"PARLIAMENT."

| Names. | Jan. 30. | Totals. |
|---|---|---|
| Holland | 62 | 209 |
| Red Cape | 38 | 132 |
| Nurse Marie | 42 | 164 |
| Garfield | 39 | 200 |
| Midget | 48 | 208 |
| E. M. S. | — | 149 |
| Sister | 59 | 203 |
| Duchess | 47 | 86 |
| Ellice | 65 | 227 |
| Reldas | 69 | 237 |
| Jenny Wren | 66 | 223 |
| Mopus | — | 122 |
| Neptune | — | 118 |
| Qu'appelle | 55 | 220 |
| Whitehoe | 64 | 211 |

| Names. | Jan. 30. | Totals. |
|---|---|---|
| Nurse Clague | — | 40 |
| Lauriston | 64 | 239 |
| B. E. T. | 36 | 141 |
| Jacko | — | 110 |
| Lady Clara | 58 | 219 |
| Patience | 68 | 239 |
| Ecila | 68 | 228 |
| Maude | 65 | 230 |
| Esperance | 69 | 234 |
| Ruby | 64 | 230 |
| M. W. | 69 | 234 |
| Crystal Palace | 60 | 182 |
| Jackanapes | 49 | 159 |
| M. M. M. | — | 25 |

**Notice to Correspondents.**

N.B.—All letters referring to this page which do not arrive at 140, Strand, London, W.C., by the *first post on Thursdays*, and are not addressed PRIZE EDITOR, will in future be disqualified and disregarded.

